# Enhanced mesoscale climate projections in TAR and AR5 IPCC scenarios: a case study in a Mediterranean climate (Araucanía Region, south central Chile)

**DOI:** 10.1186/s40064-016-3157-6

**Published:** 2016-09-28

**Authors:** R. Orrego, R. Abarca-del-Río, A. Ávila, L. Morales

**Affiliations:** 1 Departamento de Suelos y Recursos Naturales, Facultad de Agronomía, Universidad de Concepción, Concepción, Chile; 2 Scientific and Technological Bioresource Nucleus, Universidad de La Frontera, Temuco, Chile; 3 Departamento de Geofísica, Universidad de Concepción, Concepción, Chile; 4 Centro de Excelencia de Modelación y Computación Científica, Universidad de La Frontera, Temuco, Chile; 5 Departamento de Ciencias Ambientales y Recursos Naturales, Universidad de Chile, Santiago, Chile

**Keywords:** Rainfall climatology bias correction, Climate change projections, ENSO influence, IPCC TAR and AR5

## Abstract

Climate change scenarios are computed on a large scale, not accounting for local variations presented in historical data and related to human scale. Based on historical records, we validate a baseline (1962–1990) and correct the bias of A2 and B2 regional projections for the end of twenty-first century (2070–2100) issued from a high resolution dynamical downscaled (using PRECIS mesoscale model, hereinafter DGF-PRECIS) of Hadley GCM from the IPCC 3rd Assessment Report (TAR). This is performed for the Araucanía Region (Chile; 37°–40°S and 71°–74°W) using two different bias correction methodologies. Next, we study high-resolution precipitations to find monthly patterns such as seasonal variations, rainfall months, and the geographical effect on these two scenarios. Finally, we compare the TAR projections with those from the recent Assessment Report 5 (AR5) to find regional precipitation patterns and update the Chilean `projection. To show the effects of climate change projections, we compute the rainfall climatology for the Araucanía Region, including the impact of ENSO cycles (El Niño and La Niña events). The corrected climate projection from the high-resolution dynamical downscaled model of the TAR database (DGF-PRECIS) show annual precipitation decreases: B2 (−19.19 %, −287 ± 42 mm) and A2 (−43.38 %, −655 ± 27.4 mm per year. Furthermore, both projections increase the probability of lower rainfall months (lower than 100 mm per month) to 64.2 and 72.5 % for B2 and A2, respectively.

**Electronic supplementary material:**

The online version of this article (doi:10.1186/s40064-016-3157-6) contains supplementary material, which is available to authorized users.

## Background

Future climate changes will affect agriculture, hydrology, and other socio-economic fields (Caldeira and Rau 2000; Mearns 2001; IPCC 2007a, b). Atmosphere Ocean Global Climate Model (AOGCM) scenarios enable policy makers to develop new environmental strategies and mitigation methods (IPCC 2007a, b). Several different prospective scenarios are projected based on assumptions of population growth, environmental policies, technological growth, social inequality, and globalization (SRES 2000). Two scenarios for representing high CO_2_ emissions (A2) and moderate CO_2_ emissions (B2) (IPCC 2007a, b) are used as technical research in order to support public policies (e.g. Räisänen et al. 2004; RupaKumar et al. 2006; Fuenzalida et al. 2006; Conde et al. 2011; Krüger et al. 2012). These are included in the IPCC Third Assessment Report (TAR) and the Assessment Report 4 (AR4) and updated in the Assessment Report 5 (AR5), released between September 2013 and November 2014 (IPCC 2015).

Although physical laws driving the atmospheric–oceanic circulation are well-identified and the global-scale boundary conditions for modeling are highly precise and well established (Collins 2007; Räisänen 2007), climate models have different error sources (Räisänen 2007; Baigorria et al. 2008; Challinor et al. 2009). Furthermore, AOGCM were developed for global conditions (Zorita 2000), and they produce low-scale resolution climate projections (about 200–300 km). Statistical and dynamical downscaling techniques (Zorita 2000; Wilby et al. 2004) are used to improve these projections at a higher-resolution (20–50 km) over specific zones (domains) Some mesoscale projections performed are: CREAS (Regional Climate Change Scenarios for South America) in Argentina, Uruguay, and Brazil (Marengo and Ambrizzi 2006), “Variabilidad Climática para el Siglo XXI” is performed in Chile (Fuenzalida et al. 2006), and PRUDENCE over Europe (Déqué et al. 2005). In the last time was developed the CORDEX as an international effert for developing high resolution grids (Giorgi et al. 2009). Downscaled datasets inherit AOGCM uncertainties, and we should include them in order to design climate-change adaptation strategies.

On the other hand, we also should consider climate variability. For example, El Niño Southern Oscillation (ENSO) (Aceituno 1998; Vuille and Garreaud 2011) is one of the main phenomena affecting climate variability. This phenomenon affects the Pacific Anticyclone, which is the main barrier to fronts producing rain in Chile (Garreaud et al. 2009). Southern oscillation is a temporal pattern and is the difference between the measured pressure in two places: Darwin (Australia, 12°27′S, 130°50′W) and Papetee (Tahiti, 17°32′S, 140°34′W). In normal conditions, Papetee shows higher pressures than Darwin; however, this relationship is reversed under El Niño conditions (Kiladis and van Loon 1988; Guevara-Díaz 2008). Moreover, La Niña is the increase in pressure difference between Papetee and Darwin, matching with a decrease in sea temperature in coastal Chile (Kiladis and van Loon 1988). Thus, three phases of ENSO are defined: La Niña, Neutral, and El Niño phases. Nonetheless, ENSO is not the only phenomenon related to climate variability. Mantua et al. (1997) described a Pacific Decadal Oscillation (PDO) consisting of coherent interdecadal covariability in the dominant pattern of North Pacific pressure patterns and sea surface temperature. PDO can modulate the interannual ENSO-related global teleconnections (Krishnan and Sugi 2003; Wang et al. 2008) and their combined effect modulates a large part of hydrological variability within continents (Andreoli and Kayano 2005; da Silva et al. 2011; Vuille and Garreaud 2011; Wang et al. 2014). However, although ENSO is not explicitly represented in long-term projections from AOGCM (Räisänen 2007; Tebaldi and Knutti 2010; Van Haren et al. 2013), La Niña and El Niño synoptic conditions are observed. Assessing the rainfall pattern under neutral-ENSO phases allow us to understand climate variability under normal conditions, which is the basis for designing climate change mitigation countermeasures. Notwithstanding, since La Niña and El Niño conditions are not a typical pattern, it is necessary to study whether climate models represent climate variability during these phases. Since climate projections include ENSO-equivalent synoptic conditions, we can compare projections with current synoptic conditions, thus helping us to understand future climate conditions.

Our case study is focused on a Mediterranean climate, and Chilean data were used. We investigate precipitation variability within the Araucanía Region (Chile; 37° to 40°S and 71° to 74°W), which presents a very homogeneous climate associated with the Pacific anticyclone. The Pacific anticyclone produces weather conditions characterized by an important decrease in rainfall during the summer months, coinciding with higher annual temperatures (Armesto et al. 2008). The first Chilean mesoscale downscaling was computed by the Universidad de Chile’s Department of Geophysics (hereinafter DGF), with a dynamical downscaling of Hadley Centre Coupled Model (HadCM3) output (2.5° × 3.75° latitude by longitude, Pope et al. 2000; Gordon et al. 2000), using the PRECIS model (providing regional climates for impacts studies, see http://www.metoffice.gov.uk/precis/). This consisted of downscaling both the baseline data (between 1961 and 1991), together with B2 and A2 scenarios (between 2070 and 2100) at 0.25° × 0.25° resolution throughout Chile (see Fuenzalida et al. 2006 for main details and results of the experiment). HadCM3 projections are included in the IPCC Third Assessment Report (TAR, Fuenzalida et al. 2006). We refer to these downscaled fields as DGF-PRECIS.

Our goal is to first define a methodology to construct a precise, high-resolution climatology of the rainfall variability within a region under different ENSO phases and to assess its spatial variability. This initial analysis allows us to construct a database with which we can correct the data from projections, subsequently allowing us to measure the severity of future changes. We detail the steps to evaluate and correct both climate projections (TAR and AR5). In addition, several authors reported that ENSO changes extreme event frequency (Jaksic 1998; Grimm and Tedeschi 2009). Within the study zone, we construct rainfall histograms to measure frequency of rainy/dryer months, and we evaluate the statistical significance of ENSO event impacts on rainfall (one-way ANOVA test with a 95 % significance level through a Monte Carlo analysis). Although DGF-PRECIS is an important progress for assessing the effects of climate change, there are at least three issues left to be solved: (a) DGF-PRECIS dataset have been not validated with in situ data, (b) the effect of ENSO on the projected variability has not been quantified, and (c) the last IPCC report (AR5) offers new scenarios (Moss et al. 2010), while the differences between these new projections (RCP 25, RCP 45, RCP60 and RCP85) and the old A2 and B2 projections (from TAR) within our regions are still unknown. To be consistent with the original HadCM3 model, we use the Hadley model outputs included in the IPCC AR5 simulations, called HadGEM (Data distribution Center of IPCC, DDC 2015; http://www.ipcc-data.org/sim/gcm_monthly/AR5/WG1-Archive.htm). Next, we compare the DGF-PRECIS baseline database (between 1961 and 1991) with in situ data, specifically focusing on different ENSO conditions, validating the downscaled fields, and identifying possible limitations of the projected fields over the twenty-first century. Based on this comparison, we generate a corrected projection for the A2 and B2 climate change scenarios. Subsequently, we also validate and correct the new AR5 projected fields scenarios using the HadGEM simulations (Jones et al. 2011; Baek et al. 2013). Finally, we compare the TAR B2 scenario with the RCP 45 scenario and the TAR A2 with the RCP85. Additionally, we present results of the RCP 25 scenario currently used as the ideal scenario (see Table 3).

This paper is structured as follows. In “Methods” section, we present the in situ data used to construct the climatology, and we describe the model dataset used to project the rainfall condition up to the twenty-first century. Next, we discuss the methodology to study the effect of ENSO cycles on both in situ data and the DGF-PRECIS baseline. Moreover, we present statistics to evaluate the statistical significance of ENSO changes and effects in in situ fields. Correction methods and the comparison with AR5 scenarios are also presented. We present the results in “Results and discussion” section: the climatology, ENSO impact evaluation, the TAR high-resolution dynamical downscaled projection, validation and correction, and the comparison with AR5 scenarios. Finally, we discuss the results and draw our main conclusions in last section.

## Methods

### Database

**Fig. 1:**
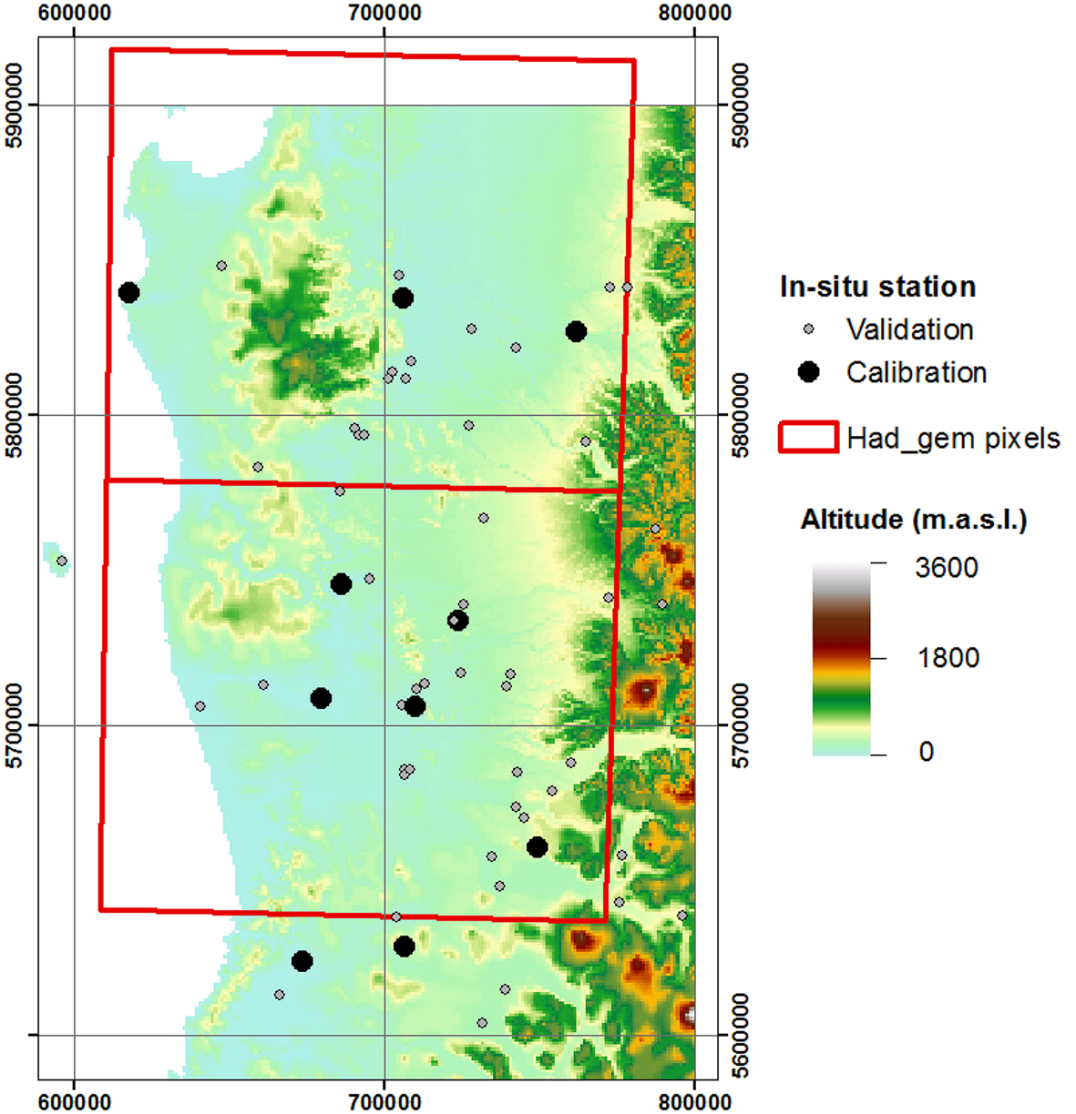
Meteorological station network overlapped over a Digital Elevation Model of the region. HadGEM pixels are *red boxes*

We used 56 meteorological stations located across the region with complete rainfall records from 1961 to 2010. In order to study projections, we focused on 1961–1991 according to the time span of the baseline of DGF-PRECIS. The baseline data were provided by *Dirección General de Aguas* (DGA, Government agency responsible for the management and administration of water resources, see www.dga.cl/) and from *Dirección Meteorological de Chile* (DMC, Government agency managing the meteorological data to predict the weather and climate in the country, see http://www.meteochile.gob.cl/). To avoid problems in the validation/correction procedure, we selected meteorological stations that fully represent the climate variability corresponding to 10-year continuous precipitation records or 15-year non-continuous precipitation records between 1961 and 2010. Based on these criteria, we selected ten stations to calibrate the DGF-PRECIS database, and the other stations were used to validate our results (Fig. 1). To complete the 15-year non-continuous records, the extended discrete Fourier transformation was used (Zhang et al. 2008). To check the data quality, we used the double mass curve method (Searcy and Hardison 1960). This method is based on a regression model between the accumulated rainfalls at two nearby meteorological stations. This approach has been successfully used in several studies, including its use to validate Galician rainfall records (Mirás-Avalos et al. 2009) and to develop ecologically relevant hydrological indexes by the United States Geological Service (USGS; Esralew and Baker 2008). Thus, in order to secure a database independent of the calibration method (Piani et al. 2010), we split into two databanks: (a) calibration database (10), built from the ten meteorological stations (held and quality controlled by DMC) with a correct geographical coverage and validated by double mass curves, and (b) validation database (46), build from the remaining meteorological station (mostly held and quality controlled by DGA).

Based on these ten meteorological stations selected, we generated a climatology to describe regional rainfall. We used climate projections based on climate change scenarios defined by several assumptions described in the six scenarios of the Special Report on Emission Scenarios (SRES 2000). Categorized by the final expected warming, these are called: A1F1, A2, A1B, B2, A1T, and B1. A 6 °C difference takes place between the most extreme scenarios, A1F1 and B1. On the focus region, most studies use only two scenarios for 2100: severe impact, A2 (850 ppm CO_2_ eq and global temperature increase 3 °C), and moderate impact, B2 (621 ppm CO_2_ eq and global temperature increase 1 °C) (Fuenzalida et al. 2006; Marengo and Ambrizzi 2006). The last IPCC Assessment Report 5 (AR5) includes new, improved scenarios based on a new concept: representative concentration pathways (RCP), corresponding to patterns which represent the expected GHG temporal behavior (Moss et al. 2010). The main scenarios are summarized in Table 1.

**Table 1:** Scenarios used in the AR5 IPCC projection (adapted from Moss et al. 2010)

Scenario	Description	Severity
RCP85	Rising radiative forcing pathway leading to 8.5 W m^−2^ (~1370 ppm CO_2_ eq) by 2100	Extreme
RCP60	Stabilization without overshoot pathway to 6 W m^−2^ (~850 ppm CO_2_ eq) at stabilization after 2100	High (comparable to A2)
RCP45	Stabilization without overshoot pathway to 4.5 W m^−2^ (~650 ppm CO_2_ eq) at stabilization after 2100	Medium (comparable to B2)
RCP26	Peak in radiative forcing at ~3 W m^−2^ (~490 ppm CO_2_ eq) before 2100 and then decline (the selected pathway declines to 2.6 W m^−2^ by 2100)	Low

Since the CMPI5 projection does not distribute the RCP 60 scenario (IPCC-data), we only analyzed the extreme scenarios available: A2 and RCP85. Additionally, we uses RCP26 as a reference, which is the lowest available concentration scenario. The low resolution HadCM3 database uses the original variables for HadCM3 (http://cera-www.dkrz.de/WDCC/ui/Index.jsp), while DGF-PRECIS variables were directly obtained from the DGF-PRECIS (the downscaled projection which was corrected) original developers. The new HadGEM fields were obtained from the IPCC data distribution center (DDC, http://www.ipcc-data.org/).

### Climatology of ENSO effects and statistical assessments

#### ENSO (Neutral, El Niño, La Niña) classification and impacts

Climatology is the monthly mean of all observations, representing a seasonal climatology which considers all possible meteorological effects. Instead of limiting ourselves to a single climatology, we used three ENSO phases: Neutral (no ENSO), El Niño, and La Niña. To assess the impact of ENSO cycles, we defined the ENSO phases by observed and simulated conditions. We used the ENSO3.4 index, defined by the sea surface temperature (SST) recorded between 120 and 170 W and 5N–5S. This index has a significant correlation with the climate in South America (Jaksic 1998; Garreaud and Battisti 1999; Rigozo et al. 2004). We started classifying the in situ data and DGF-PRECIS output according to IRI criteria (see Guevara-Díaz 2008): each month was classified as La Niña, El Niño or Neutral (http://iridl.ldeo.columbia.edu/). SST over the Equator were downloaded from the HadCM3 baseline (1961–1991) scenario (available at http://www.ipcc-data.org/) to construct a corresponding IRI criteria ENSO3.4 index and to classify the DGF-PRECIS output according to ENSO cycles.

ENSO impacts on climate rainfall condition were evaluated by comparing the monthly climate average of each ENSO phase over the time span (1961–2010) for in situ data fields and DGF-PRECIS fields (1961–1991). These phases were also mapped in order to identify the spatial pattern of the ENSO effect on both datasets. Mapping was performed through ordinary krigging spatial interpolation of in situ climatology for each ENSO phase (Isaaks and Srivastava 1989).

Since climatology does not represent extreme events such as highly wet or dry months, we computed rainfall histograms over the whole baseline period (1961–2010) for the whole in situ dataset, classifying them into ENSO phases. We grouped these into 25 mm intervals and considered the “no rainfall” case as a separate group. This selection helped us to evaluate the effect of ENSO on the number of months without rainfall events.

#### Statistical assessment

We evaluated the statistical significance of the impact of ENSO rainfall events using a one-way ANOVA test with a 95 % significance level. When the differences are significant, variance between ENSO phases should be higher than the variance of the phase. The rate between both values follows a Fisher distribution F, allowing us to evaluate the significance of the relationship. Thus, a significant difference among ENSO conditions takes place when this rate is higher than the critical F value (Wilks 2006). To perform an even more robust test, we used a Montecarlo analysis, consisting of fitting a stochastic model based on the observed data to produce a syntactical data series following the same probabilistic distribution as the observed data. 10,000 rainfall data were generated based on Weibull distribution, fitted for each ENSO-condition using the in situ database. Finally, the ANOVA test was performed using the syntactical data instead of the observed data.

### Comparison between meteorological records and DGF-PRECIS Outputs

We downloaded the downscaled fields from the data system management of “Variabilidad Climática en Chile” project (http://www.dgf.uchile.cl/~maisa/modelacion_climatica/), and we selected the grid values corresponding to each meteorological station. It was not possible to compare the DGF-PRECIS baseline (1961–1991) one by one with its corresponding (1961–1991) in situ measurement since climate models produce results in the form of projections rather than forecasts (Wood et al. 2004; Knutti 2008). Consequently, we compared them by global statistics and histogram analysis, considering the whole validate database. Residual and spatial analyses were also performed by mapping in situ and projected annual cumulative precipitation using ordinary Krigging techniques (Isaaks and Srivastava 1989). Both evaluations were initially carried out using the whole database and separating ENSO (El Niño, La Niña) and non ENSO (Neutral) conditions.

### TAR (DGF-PRECIS) correction and AR5 projections

We proposed two methods in order to perform a bias correction: correction based on coefficient rates (CBCR) and correction based on quantile mapping (CBQM), explained in the following:

- Correction based on coefficient rates (CBCR) between simulated and measured monthly records of precipitation (Eq. 1).
  1 $${fr_{m} = \frac{{rm_{is} }}{{re_{m} }}fe_{m} }$$

where *fr*_*m*_ is the corrected projection, *rm*_*is*_ is the monthly average of in situ data, *re*_*m*_ is the monthly average of estimated data, and fe_m_ is the DGF-PRECIS data. It is important to state here that we generate the monthly coefficient *rm*_*is*_*/re*_*m*_, based on the calibration database (that is based into 10 meteorological stations, see “Database” section). Since climate models represent global trends, this coefficient was computed using all the in situ data. A global coefficient (unique for the whole image) is obtained for each averaged month. The final product is a DGF-PRECIS dataset without bias.

Correction Based on Quantile Mapping (CBQM) corrects the RCM-simulated precipitation based on constructed empirical cumulative distribution functions (ECDF). The frequency of precipitation occurrence is corrected at the same time (Chen et al. 2013). Thus, the corrected RCM is computed by the following equation (Eq. 2):

2 $${fr_{m} = ECDF_{is}^{ - 1} (ECDF_{rm} (\text{Re}_{m} ))}$$

where *fr*_*m*_ is the corrected projection, $ECDF^{ - 1}_{is}$ is the inverse of the empirical cumulative distribution functions of the in situ data, *ECDF*_*rm*_ is the empirical cumulative distribution functions of the modeled data, and *Re*_*m*_ is the DGF-PRECIS data. The empirical cumulative distribution functions were determined for each month and are based on the Weibull distribution. In this case also, the model was computed using the calibration database.

The corrected dataset based on both methods was compared with the validation database by histograms in order to choose the best fit respect to database histogram, which was defined based on Euclidean distance. Thus the most fitted was that it shows less Euclidian Distance with in situ data.

The corrected dataset based on both methods was compared by histograms with the validation database in order to choose the best fit respect to database histogram, which was defined based on Euclidean distance. Thus, the chosen fit is the one presenting lesser Euclidian Distance with the in situ data.

Based on these corrected scenarios, we assessed the impact of climate change on rainfall by computing climatology and histograms of the precipitation intensity such as in the case of the baseline in situ data. Histograms were computed to quantify the effect of climate change on extreme events (very high precipitation or very dry months), which has been previously reported by various authors in South America (Marengo et al. 2009).

Lastly, we compared these corrected projections with the AR5 projections. To perform a consistent comparison, we used the HadGEM projections following the same bias correction method used for DGF-PRECIS and AR5 projection. The baseline was extracted from the historical runs over the HadCM3 time span (1961–1991) and then the 2070–2100 periods were validated and corrected. AR5 projections were provided by the IPCC data distribution center (DDC). Since the region is covered by two HadGEM pixels, we divided the in situ data into northern (up to 38°15′S) and southern (down to 38°15′S) regions (pixels are 1°15′ × 1°45′ centered in 37°30′S, 72°45′W and 38°45′S, 72°45′W; Fig. 1).

The comparison was performed for the same baseline period as TAR DGF-PRECIS (1961–1991). First, we averaged all in situ records (the meteorological stations) located inside HadGEM pixels, in order to simulate the rainfall at HadGem pixel resolution (Tustison et al. 2001). Second, we compared the averaged in situ data with the HadGEM pixels, and we performed a monthly correction for the whole region (as DGF-PRECIS). This correction was applied to the AR5 projected scenarios (RCP25, RCP45 and RCP85), separating each HadGEM pixel. Third, we also averaged the corrected DGF-PRECIS cells located inside the HadGEM pixels for the whole region. Finally, the comparison was made between the corrected DGF-PRECIS and the corrected HadGEM pixel scenarios (B2 and A2 with RCP 45 and RCP 85, respectively).

## Results and discussion

### Regional climatology

**Fig. 2:**
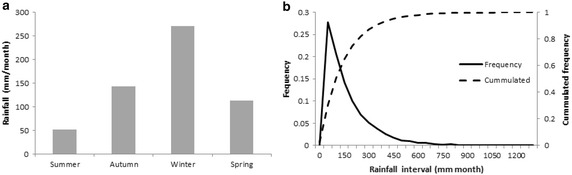
**a** Seasonal precipitation and **b** relative (*solid line*) and cumulative (*dashed line*) frequencies of monthly precipitation

Our analyses show that the Araucanía Region is characterized between 1961 and 2010 by mean annual accumulated precipitation of 1750 ± 29 mm per year. Higher rainfall levels occur during the winter of 259 mm per month, and lower rainfall levels occur during the summer of 53 mm per month (Fig. 2a). The left-skewness of the monthly precipitation curve shows that most monthly precipitation is distributed between 5 and 150 mm per month, with a median of 90 mm per month (frequency of 7 %) and a peak of 25 mm per month (27 %) (Fig. 2b). Months without rainfall occur with a frequency of 2.4 % (Fig. 2b), meaning almost never.

### ENSO effect

Since ENSO is one of the main Chilean climate drivers, we focus our description on its variability. During the research period of January 1961 to December 1991, we observed 84 La Niña months and 99 El Niño months, corresponding to 5 La Niña events and 10 El Niño events as we showed in Additional file 1. General rainfall spatial distribution patterns were similar during all ENSO conditions. Nevertheless, there are important differences among ENSO rainfall amount, which changes depending on zone and season. This is a different pattern with respect to other classified Chilean regions in the same Mediterranean climate (Rouanet 1983), where El Niño conditions imply rainy years, and La Niña conditions imply dry years (Aceituno 1998; Jaksic 1998; Garreaud et al. 2009).

**Fig. 3:**
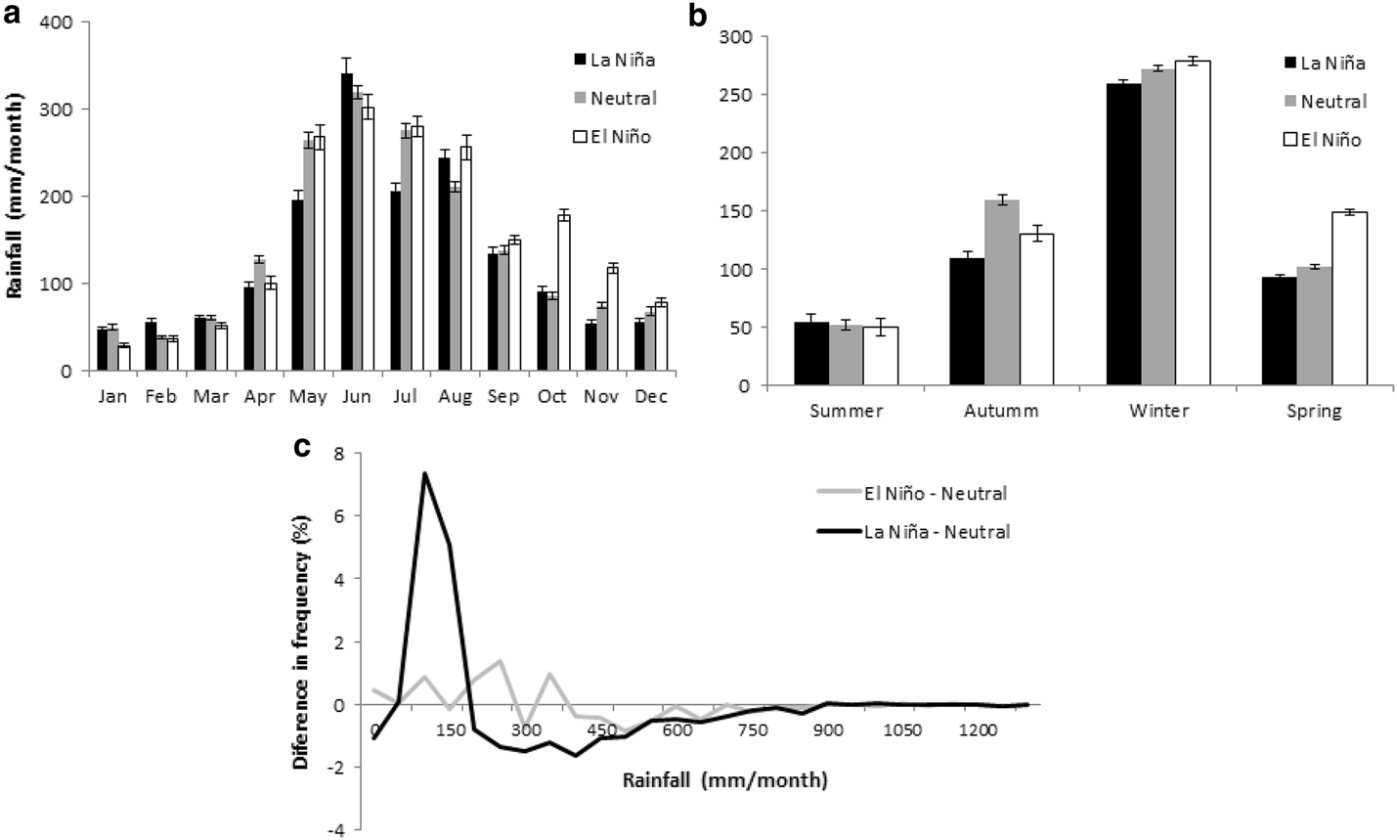
Annual rainfall cycles of ENSO scenarios **a** monthly and **b** seasonal. Differences in monthly rainfall frequencies between El Niño—Neutral and La Niña—Neutral conditions are shown in (**c**). *Error bars* correspond to standard error. Histograms were constructed based on intervals of 50 mm

During El Niño events, the mean accumulated precipitation is 1851 mm per year, that when compared with 1718 mm per year in neutral years, corresponds to a +133 mm per year increase. Under La Niña events, the mean accumulated precipitation is 1586 mm per year (Fig. 3), corresponding to a −132 mm per year decrease. Based on the monthly averages (Fig. 3a), during El Niño events, we observed a low effect with respect to La Niña. In fact, during El Niño we do not observe a significant change in the frequency of low rainfall months (lower than 100 mm per month, from 45.2 to 46.0 %), with high rainfall between 100 and 350 mm per month, from 41.3 to 43.2 %, and in the extreme months over 300 mm, from 7.8 to 10.9 % (Fig. 3c). A different pattern is observed during la Niña, where the number of high rainfall months increases from 45.2 to 57.8 %, the number of low rainfall months decreases from 41.3 to 34.7 % (Fig. 3b), and the number of extreme rainfall months decreases from 10.9 to 6.0 % (Fig. 3c). Changes in “No Rainfall” events were not significant (Fig. 3c).

For the seasonal averages (Fig. 3b), during the spring months of El Niño years, precipitation increases by 46.3 %, i.e. +47 mm per month in comparison to neutral years, while during the autumn months of El Niño years, precipitation decreases by 17.9 % (−28.5 mm per month) in comparison to neutral years. On the other hand, in La Niña years, we observe a significant decrease in autumn (30.9 % or −49 mm per month), but no significant changes in other seasons (9.0 % or less). These trends confirm the ANOVA test of the Montecarlo analysis outputs, showing that the variance explained by each ENSO condition and total variance is higher than the critical F values. Therefore, statistically significant differences among ENSO conditions take place.

Rainfall spatial distribution patterns are similar during all ENSO conditions, although a small decrease in the latitudinal precipitation gradient occurs during El Niño and a small increase under La Niña (Fig. 6). During the winter period, La Niña events are generally characterized by an increase in the precipitation levels in the northwestern zone (37°S, 73°W) and a low increase in levels in the southeastern zone (39°S, 72°W), which is generally the region with the largest rainfall (Fig. 6). This pattern is reversed under El Niño winter events: greater precipitation levels are observed in the southeast, while lower levels are observed in the northwest.

### Evaluating DGF_PRECIS dataset

**Fig. 4:**
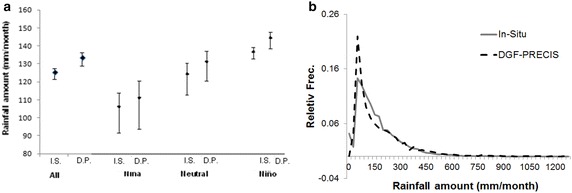
**a** Mean precipitation for In-Situ and projected by DGF-PRECIS values under all ENSO conditions and for global simulation (All). *Error bars* represent standard error (over) and 95 % significance values (under). **b** Global histogram comparing DGF PRECIS and In-Situ rainfall

Simulated mean monthly rainfalls (134 mm per month for DGF-PRECIS in 1961–1991) are higher than the in situ measured values (125 mm per month for 1961–1991), meaning that the model overestimates rainfall. This pattern is also observed during all ENSO conditions (Fig. 4a). However, error bars overlap, showing that there is no significant difference between the in situ measurements and simulated data (Fig. 4a). In fact, the difference between the means of in situ and DGF-PRECIS data is −8.9 mm, representing only less than 7 % of the amount of measured annual rainfall. Rainfall histograms show a significant overestimation of small rainfall events (lower than 100 mm), with the peak at 50 mm more prominent in the simulated curve than in the in situ measurements (Fig. 4b). This pattern is a good representation of the drizzle effect discussed by several authors (Baigorria et al. 2008; Piani et al. 2010), which is inherent in all AOGCM (Baigorria et al. 2008).

**Fig. 5:**
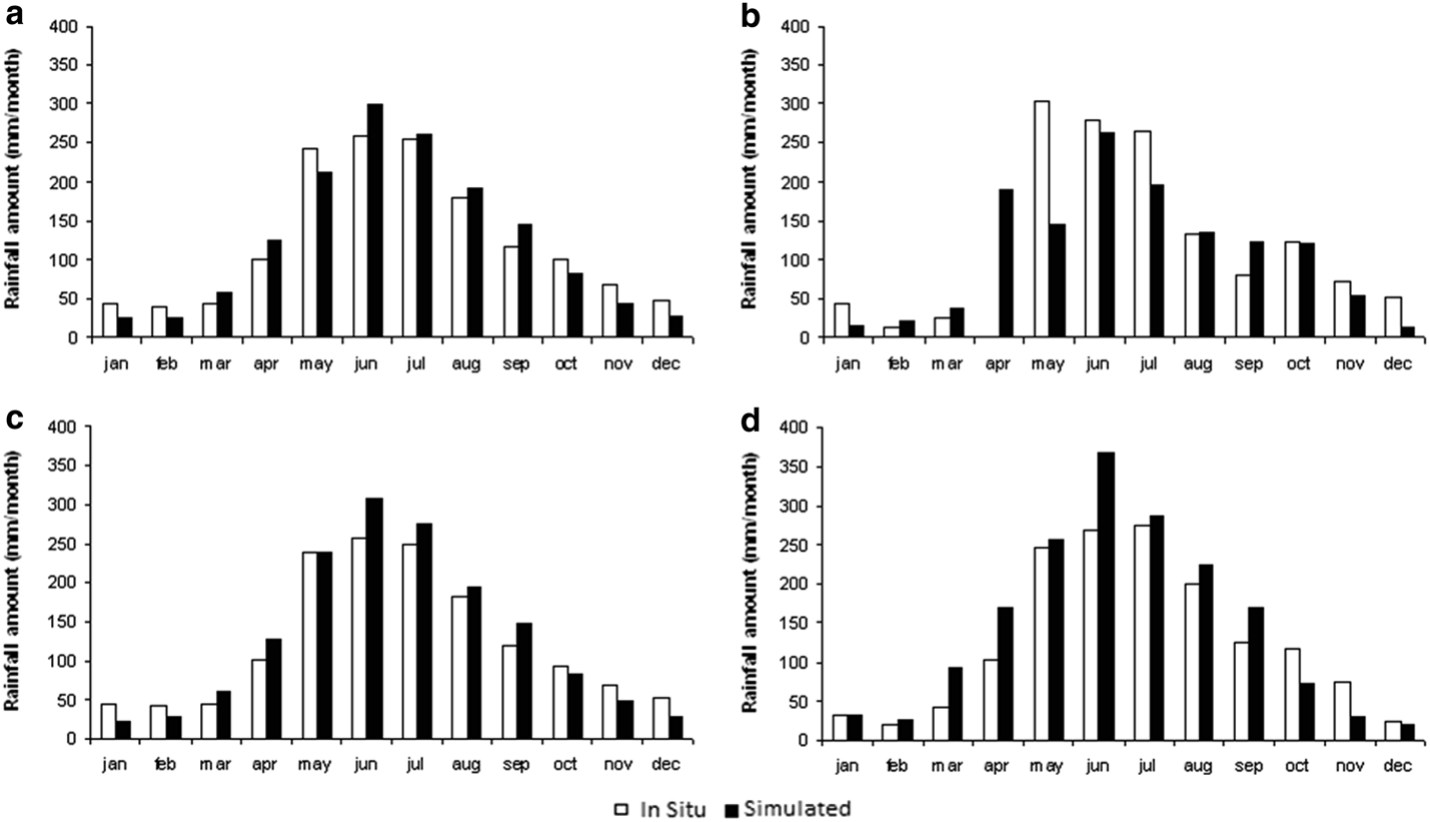
In-situ and simulated (HadCM3 downscaled by PRECIS downscaled) monthly rainfall for all-data (**a**), La Niña condition (**b**), neutral condition (**c**) and El Niño condition (**d**)

In relation to monthly rainfall distribution, DGF-PRECIS presents larger seasonal variability than the observed cycle (standard deviation of precipitation during the 1961–1991 period of 157 mm for DFG-PRECIS and 122 mm for In-situ records; Fig. 5a). In fact, simulated summer months are drier (38.3 %, i.e. −16.8 mm per month), whereas simulated winter months are wetter (15 %, i.e. +34.6 mm per month) than those measured (Fig. 5a). Under El Niño conditions (Fig. 5d), seasonal distributions of monthly rainfall are overestimated, except during the summer months. In contrast, La Niña conditions are well-estimated for February, March, June, August, and October, although there are important differences during the remainder of the year (Fig. 5b).

**Fig. 6:**
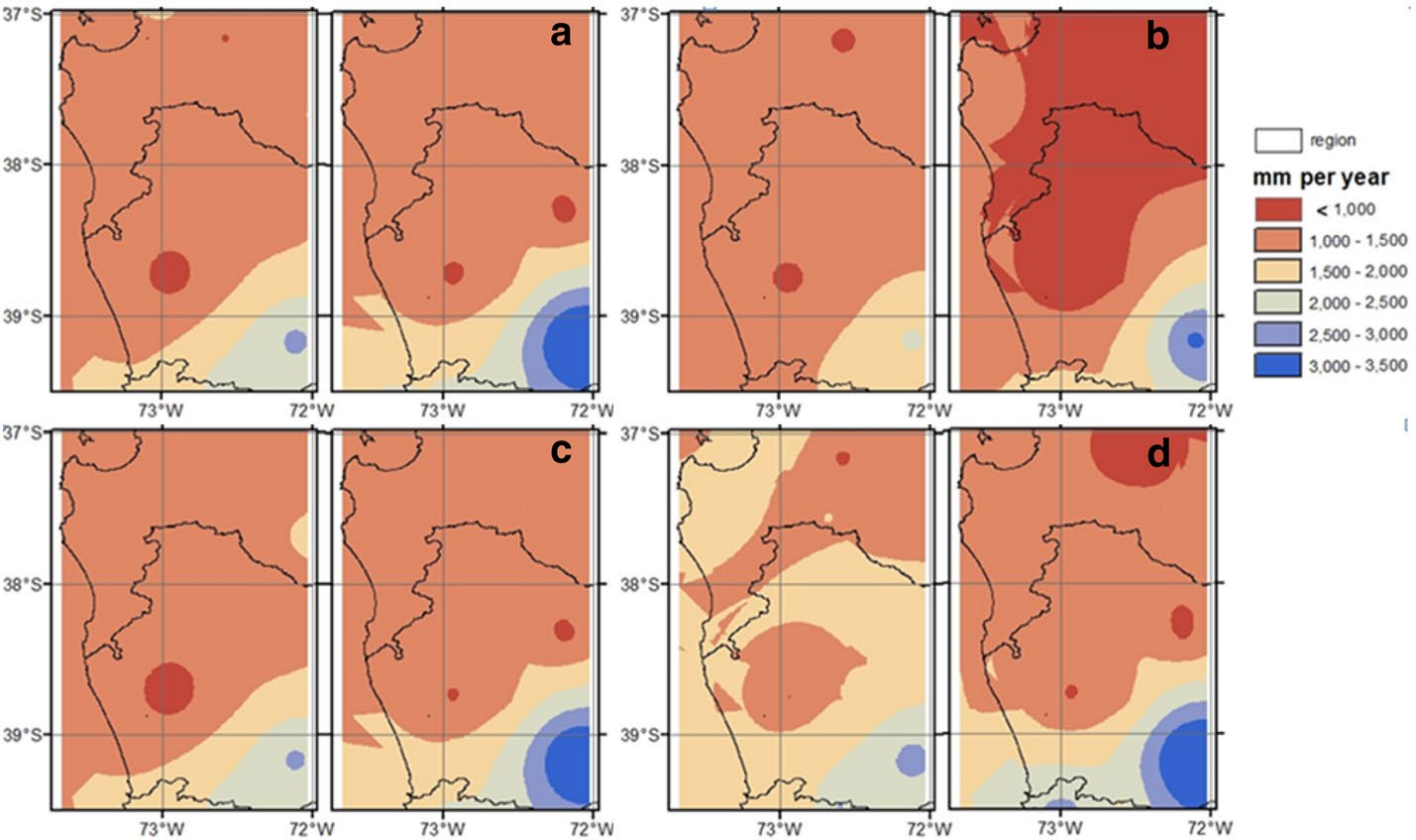
Spatial patterns of annual accumulated precipitation of measured (in situ) (*left*) and HadCM3 downscaled by PRECIS projection over the base line period (1962–1991) (*right*), considering **a** Climatology, **b** La Niña condition, **c** Neutral condition and **d** El Niño condition

From a geographic point of view, a positive precipitation gradient from the northwest to the southeast is observed at a rate of 4.78 mm km^−1^ (Fig. 6). The largest rainfall amount is observed in the southeast (39–39.5°S, 72.5–71.5°E) about 2500 mm per year, and the lowest rainfall amount of about 800 mm per is observed in the west coast (37–37.5°S, 73–73.50°E). HadCM3 downscaled by PRECIS underestimates rainfall levels in the north (by about 10 %, i.e. −10 mm per month) and overestimates them in the south (by about 30 %, i.e. +50 mm per month) for neutral conditions. A similar pattern occurs during El Niño and La Niña years, where the model underestimates northern and overestimates southern rainfall rates by about the same values (Fig. 6b–d).

### Correction of the baseline and projected climatology

PRECIS shows unsystematically biased estimates when reproducing ENSO effects on rainfall (“Evaluating DGF_PRECIS dataset” section). Thus, we do not construct a corrected projection for the different ENSO phases, but exclusively for general conditions, i.e. without ENSO discrimination.

**Fig. 7:**
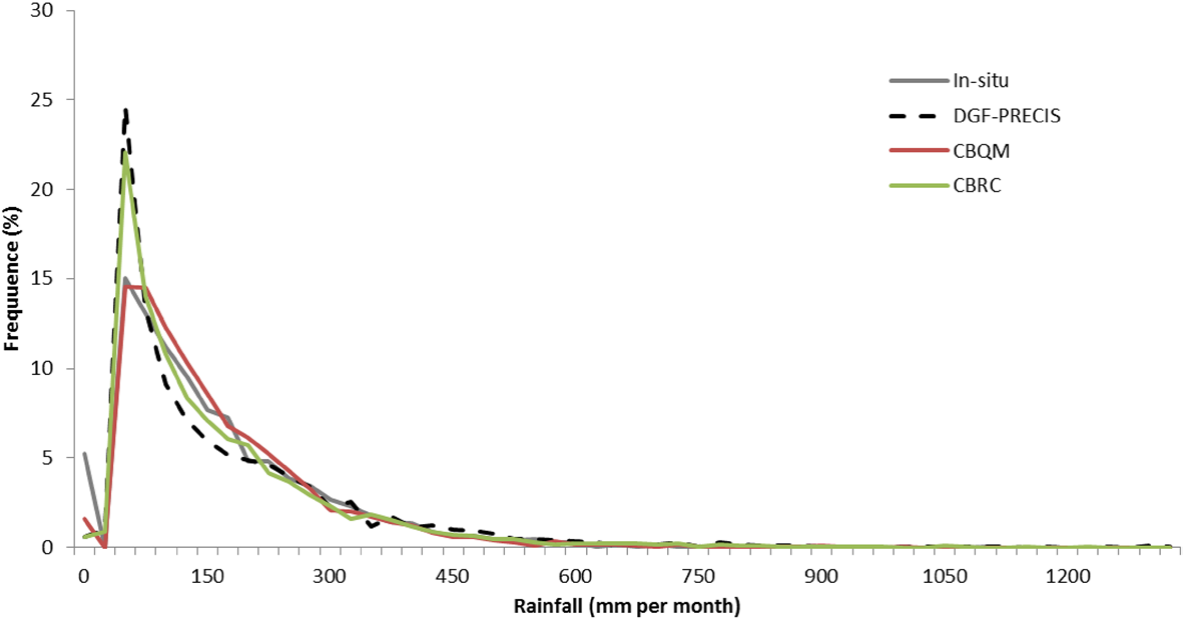
Histogram of modeled, in situ, and corrected (quantile mapping and coefficient rates) rainfall

We compared both bias correction methods to evaluate which would afford us a better estimate of what might occur in the future. Grounded on this comparison, quantile mapping produces the most fitted results, indicating a better Euclidean distance (and therefore better visual balance) of histograms with respect to in situ data: 23 mm (Fig. 7). These results are in line with other authors who compare different methods of bias correction and report significant improvement in RCM performance by using quantile mapping (Chen et al. 2013; McGinnis et al. 2015; Fang et al. 2015). Even so, we report here the consequences of both methods so as to show that even though one is plainly more adept than the other, the two are corroborated in the sense of what the future of rainfall in the region may be.

**Fig. 8:**
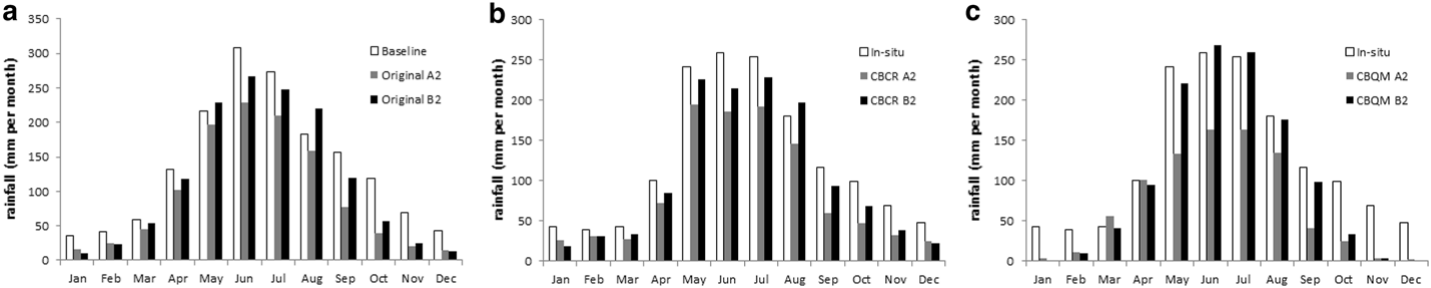
Original and Corrected monthly mean rainfall values (1962–1981), A2 and B2 scenarios: **a** Baseline, **b** In-situ and CBCR and **c** In-situ and CBQM

Both corrected scenarios show a reduction in annual precipitation, which is higher under A2 (a reduction of 30.4 %, i.e. −458 ± 42. 4 mm per year for CBCR and 43.8 %, i.e. −655.9 ± 27. 4 mm per year for CBQM) than under the B2 scenario (15.5 %, i.e. 235 ± 87 mm per year for CBCR and 19.19 %, and −287 ± 42 mm per year for CBQM). In both scenarios and cases, the corrected values represent a reduction in precipitation larger than the original uncorrected projection simulation (24.3 and 7.6 %, i.e. −363.9 and −113.6 mm per year for A2 and B2, respectively); see Fuenzalida et al. 2006 (Fig. 8; Table 2).

**Table 2:** Change observed under both climate change scenarios

Month	In-situ	Original B2	CBCR B2	CBQM B2	Original A2	CMCR A2	CBQM A2	Original baseline
Jan	43.2	10.6	18.68	0.97	16	26.16	3.8	35.4
Feb	39.8	23.2	31.51	10	24.5	31.21	11.6	41.4
Mar	43.4	53.9	34.03	41.1	45.6	28	56.1	58.6
Apr	100.3	118.1	84.51	94.4	101.7	72.42	101.7	132
May	242.1	228.6	226.34	221.6	196.7	194.97	134.1	216.2
Jun	259	267.3	215.19	268.5	228.9	185.49	163.3	308.3
Jul	254.3	247.8	229.08	259.9	209.7	192.62	163.5	273.4
Aug	180.6	220.1	197.86	176.6	158.6	145.6	135.5	182.4
Sep	117.3	119.6	93.72	98.4	77.3	60.54	41	156.4
Oct	99.9	57.3	68.87	33.1	39.7	47.67	24.6	118.9
Nov	68.7	24.3	39.11	4.1	20.2	32.01	3.4	69.4
Dec	48.5	12.7	22.65	0.96	14.3	25.46	2.6	42.6
SUM	1497	1383.5	1265.34	1209.6	1133.1	1042.15	841.1	1635

Original refers to the original DGF-PRECIS downscaled rainfall (mm per month) instead of In-Situ referring to the base line rainfall (mm per month). CBCR is referred to correction based on coefficient rates and CBQM is referred to correction based on quantile mapping

**Fig. 9:**
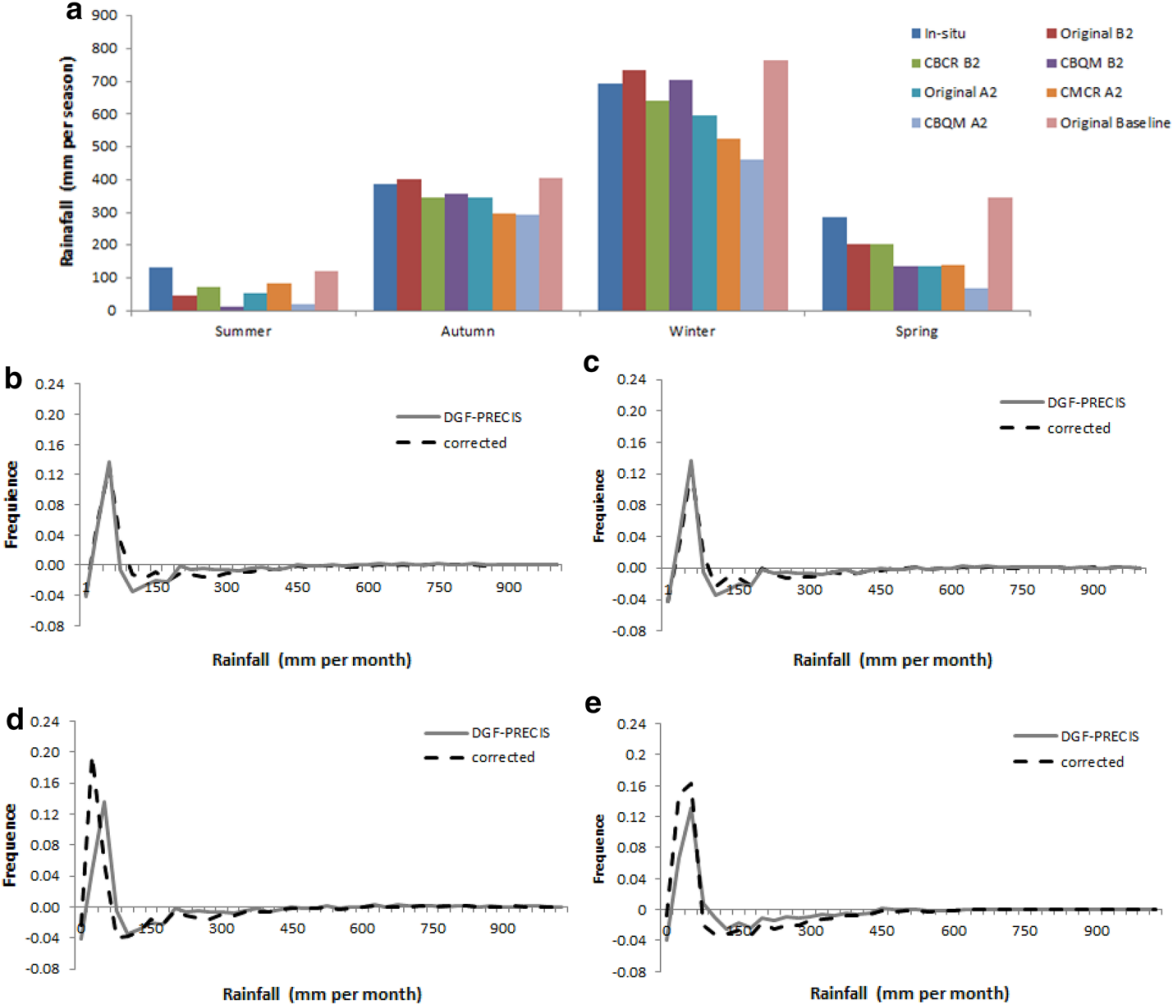
Simulated seasonal rainfall (**a**), CBCR corrected B2 difference amount frequency (base line period—projected) (**b**), CBCR A2 difference amount frequency (base line period—projected) (**c**), CBCR corrected B2 difference amount frequency (base line period—projected) (**d**) and CBQM A2 difference amount frequency (base line period—projected) (**e**)

For CBQM, the reduction in precipitation is higher for summer (86 %, i.e. −113.4 mm/season for A2; 91 %, i.e. −119.5 mm for B2) and spring (75 %, i.e. −216.9 mm/season for A2; 52 %, i.e. −150.3 for B2) than for winter (25 %, i.e. −170.1 mm/season under A2; 7 %, i.e. −51.6 mm/season for B2) and autumn (23 %, i.e. −90.3 mm/season for A2, 10 %, i.e. −37.2 mm/season for B2) (Fig. 9). Similar results are obtained when CBCR methods are used. These corrected scenarios show that the reduction in precipitation is higher for summer (37 %, i.e. −48.6 mm/season for A2; 45 %, i.e. −58.8 mm for B2) and spring (51 %, i.e. −145.8 mm/season for A2; 29 %, i.e. −84.3 for B2) than for winter (33 %, i.e. −231.5 mm/season under A2; a not significant increase of 1.6 %, i.e. +11.16 mm/season for B2) and autumn (24 %, i.e. −94.0 mm/season for A2, 7 %, i.e. −28.8 mm/season for B2) (Fig. 9; Table 2).

Although both corrected methods are consistent in projecting reductions in rainfall, CBCR projects lower reductions for autumn and winter than the original uncorrected DGF-PRECIS (11 %, i.e. −43.5 mm/season and 14 %, i.e. −102.4 mm/season for A2 autumn and winter, respectively, and 4 %, i.e. −15 mm/season and 6 %, i.e. −42 mm/season month for B2 Autumn and winter, respectively) and higher for summer and spring (52 %, i.e. −104.7 mm/season, and 58 %, i.e. −28.5 mm/season for A2 summer and spring, respectively, and 30 %, i.e. −59.7 mm/season and 65 %, i.e. −30 mm/season for B2 summer and spring, respectively) (Fig. 9a). Instead, CBQM projects higher reductions for all seasons than the original projection for A2 (11 %, i.e. −43.5 mm/season and 14 %, i.e. −102.4 mm/season for autumn and winter, respectively, 52 %, i.e. −104.7 mm/season, and 58 %, i.e. −28.5 mm/season for summer and spring, respectively), higher for summer, spring, and autumn of B2 (30 %, i.e. −59.7 mm/season and 65 %, i.e. −30 mm/season, and 6 % i.e. −42 mm/season for summer, spring, and autumn, respectively), and less winter of B2 (and 4 %, i.e. −15 mm/season) (Fig. 9a; Table 2).

Corrected projections suggest lower rainfall during the winter and autumn, which may explain the lower annual accumulated values; however, the main differences are observed in the summer. Based on the histograms, we observed that both projections increase the frequency of months with lower rainfall (less than 100 mm per month) from 57.17 to 68.52 % for A2, from 57.17 to 64.12 % for B2, respectively, (CBCR) 57.17–72.5 % for A2, and from 57.17 to 64.18 % for B2, respectively (for CBQM) during the time span (1961–1991). In both frequency analyses, we used the corrected database with respect to the in situ values (Fig. 9).

**Fig. 10:**
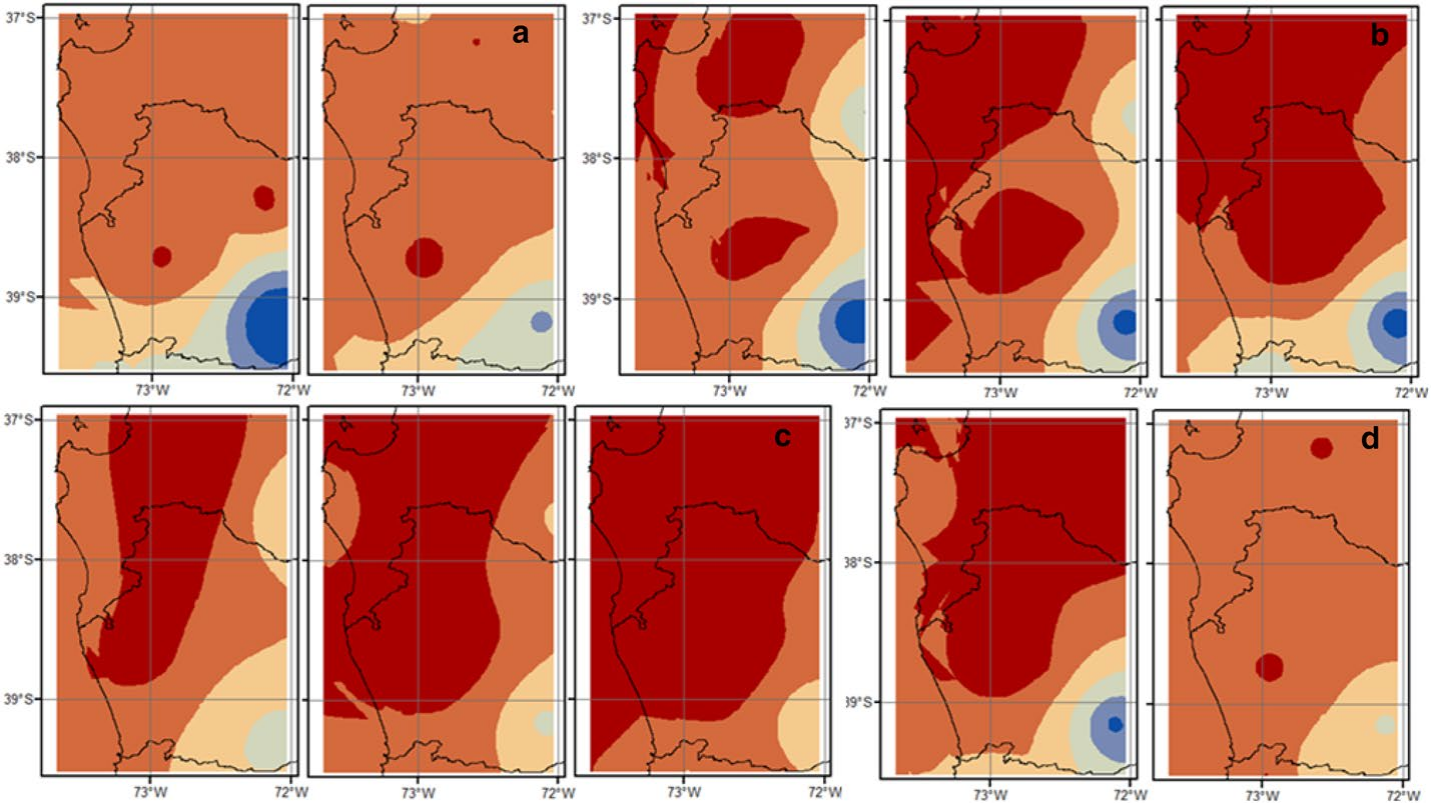
Spatial patterns of precipitation for values under **a** current (baseline epoch, 1961–1991), **b** B2 (2070–2100) and **c** A2 (2070–2100) scenarios, and **d** La Niña base line condition. In (**a**) and (**d**), *left map* shows the original PRECIS projection and *right* the corrected values. In (**b**) and (**c**) *left map* shows the original PRECIS projection, center map shows the CBCR projection and *right map* shows the CBQM projection

We notice that the spatial pattern of precipitation predicted by the climate change scenarios shows a general decrease in the whole region, but higher for the northern coastal precipitations (about 37.5°S, 73°W). This is observed regardless of the correction method, but there are differences in some places. In CBCR, an increase is observed (by about 120 mm per year under scenario A2 and 300 mm per year under B2), whereas in CBQM, this zone shows a small decrease (by about 100 mm per year) under the B2 scenario. On the other hand, CBCR shows that coastal precipitation (left side of each map) will decrease (about 700 mm per year under A2 and 400 mm per year under B2) along with CBQM, which projects a decrease by about 700 mm per year under scenario A2 and 500 mm per year under B2 (Fig. 10).

Furthermore, both correction methods shows an important decrease in the south of about 500 mm per year under A2 and an increase of 100 mm per year under B2; this increase is observed only in the mountain zone (right side of the map) in CBCR and in all zones in CBQM (Fig. 10). This area usually experiences the highest levels of precipitation and is where important agricultural centers are located (Peña and Romero 1977; INE 2010).

**Fig. 11:**
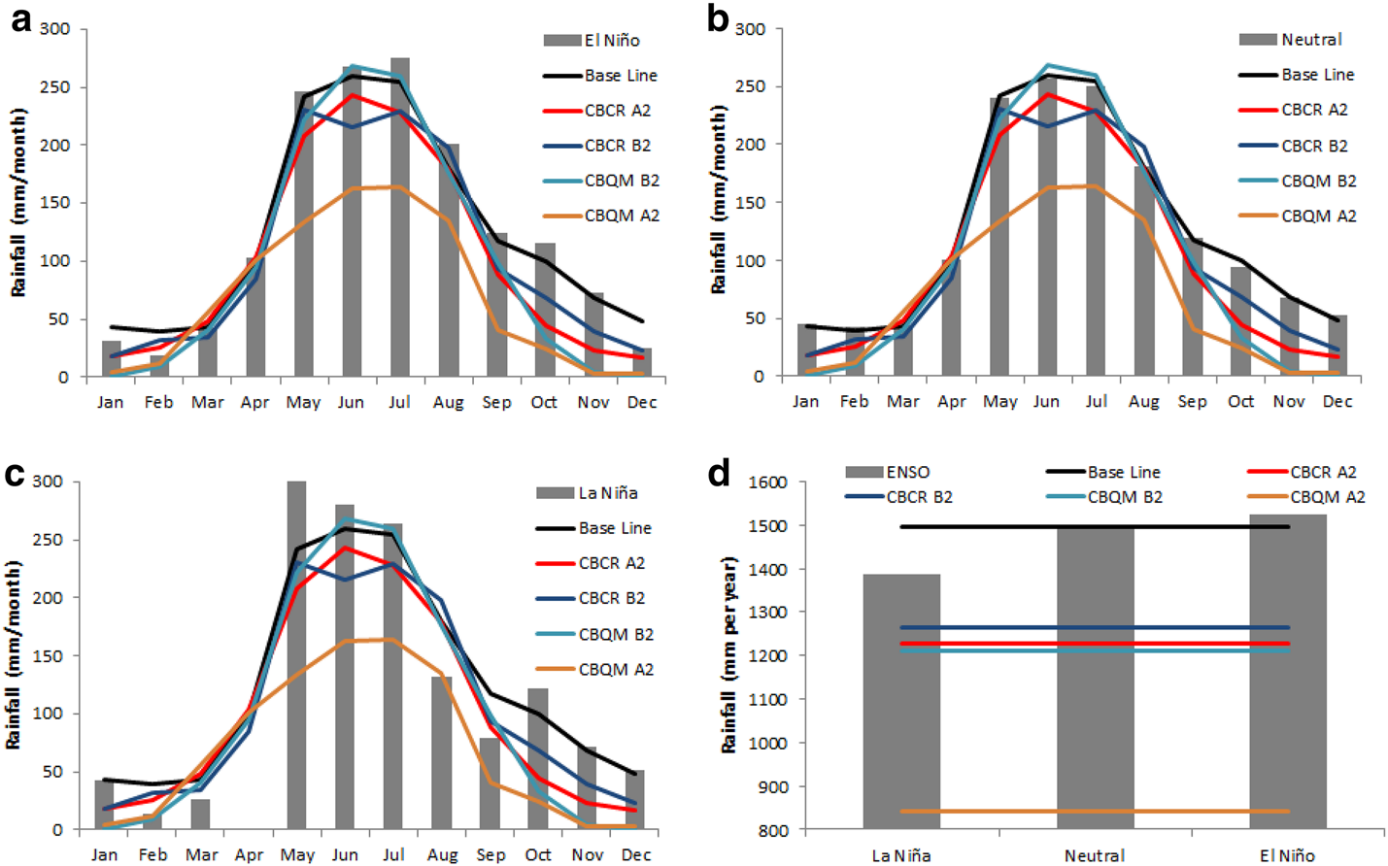
DGF-PRECIS scenarios compared with **a** El Niño, **b** Neutral (non ENSO) and **c** La Niña. **d** Yearly cumulated

We compare the climate pattern expected for the climate change scenarios with the different ENSO phases. Our results show that the expected climate patterns are closer to La Niña conditions than those observed in the neutral or El Niño phases; however, this pattern is even more extreme under the climate change scenarios, especially in spring and summer (Fig. 11). Eventually, in A2 scenarios, the final quantity of precipitation theoretically reaching the area is less than the one experienced on average during La Niña years from 1961 to 1991, which is a substantial reduction. This is regardless of the correction method, but CBQM marks the differences (Fig. 11d).

### AR5 comparison

AR5 simulations were bias-corrected by both CBCR and CBQM methods following the same methodology used for A2 and B2 scenarios (Table 3). Nonetheless, we only report the CBQM results, following a better rainfall distribution correspondence than CBCR, as both methods present analogous effects. A reduction in the rainfall amount is projected, but are there real differences between the corrected DGF-PRECIS and corrected AR5 projection scenarios? For example, let us compare AR5′s RCP45 with the TAR’s B2 scenario. If we consider the whole region, the corrected AR5 projections show similar or comparable effects to the TAR. It decreases with respect to in situ climatology by about −298.7 mm per year (−20.27 %) for AR5′s RCP45, compared to a decrease of −287 mm per year (−19.19 %) observed in the TAR’s B2 scenario. Likewise, we noticed the same when we compare A2 with RCP85 scenarios, although the corrected DGF-PRECIS shows a reduction in precipitation (−655.9 mm per year, −43.8 %) larger than the corrected RCP85 (−447.2 mm per year, −30.38 %).

**Table 3:** Changes observed under AR5 change scenarios

Month	In-situ	Original	Corrected RCP26	Original	Corrected RCP45	Original	Corrected RCP85
		RCP26		RCP45		RCP85	
Jan	43.2	26.6	25.4	20.6	21.7	19.1	13.9
Feb	39.8	41.1	39	24.5	24.9	19.8	13.4
Mar	43.4	65.1	60.2	44.2	43.4	45.6	35.7
Apr	100.3	116.5	106.2	106.2	97.7	109.5	84.6
May	242.1	272.5	244.2	218.3	192.9	149.4	122.3
Jun	259	310	277	213.9	189.8	266.0	236.4
Jul	254.3	234	210.5	248.6	216.8	246.8	217.3
Aug	180.6	176.7	159.8	143.8	129.4	153.7	127.5
Sep	117.3	135	122.7	95.5	88.6	95.7	75.5
Oct	99.9	105.9	96.7	95.9	88.9	73.4	55.9
Nov	68.7	52.6	49.2	48.2	47.2	32.0	23.2
Dec	48.5	41.2	38.8	33.4	33.7	28.7	20.4
SUM	1497	1577	1429.5	1293	1175	1240	1026

Original refers to the original AR5 rainfall scenarios before correction (mm per month), while In-Situ refers to the baseline rainfall (mm per month)

**Fig. 12:**
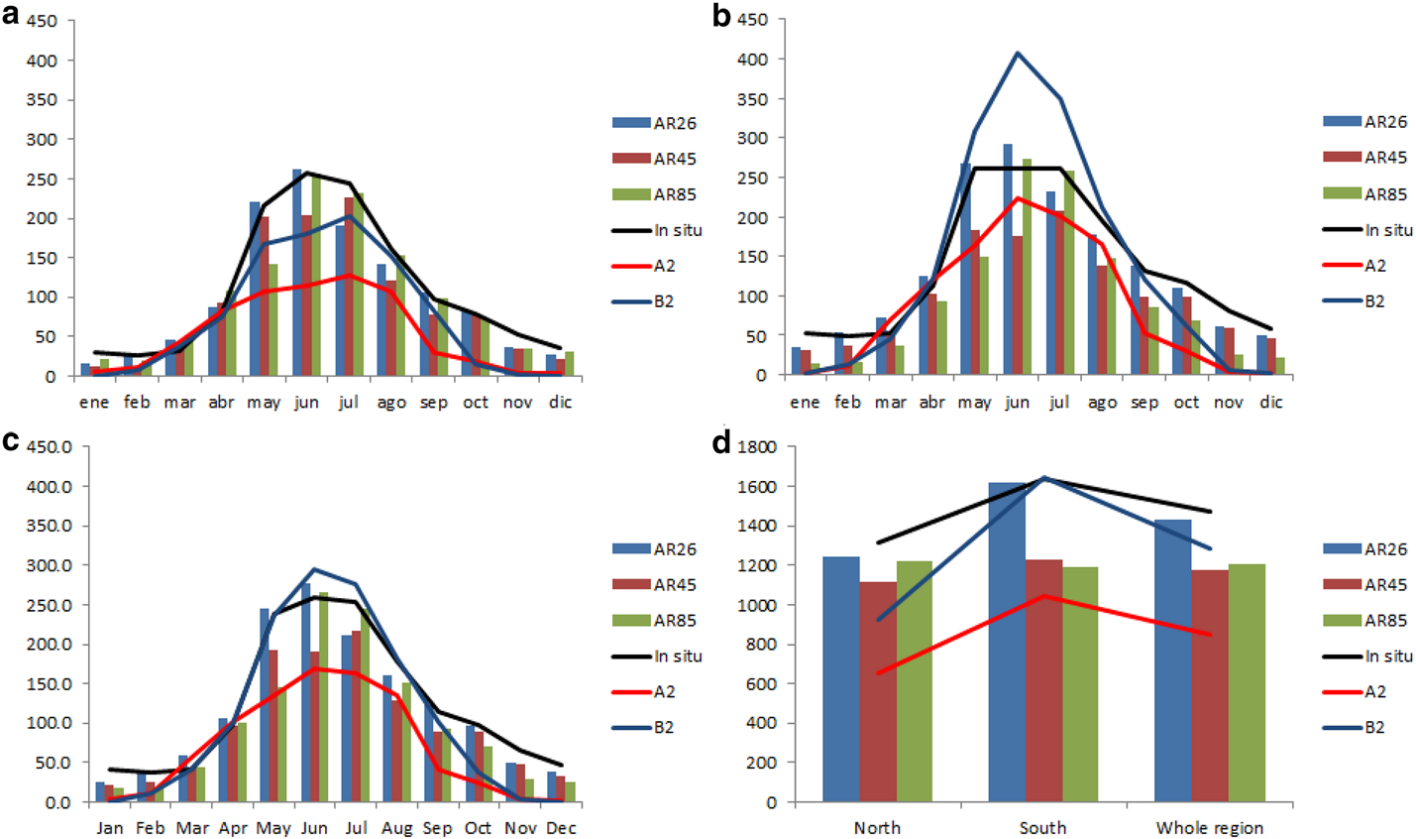
Monthly rainfall projections comparison between TAR (*lines*) and AR5 (*bars*) projections for **a** Northern region, **b** Southern region, **c** whole region and **d** whole year

AR5 also coincides with TAR as both databases project comparable effects in terms of mm/season, but lesser in terms of percentage during winter and summer. Thus, for RCP45, rainfall decreases by about 153.9 mm (−22.3 %) during the winter and only −46.1 mm during the summer, lesser in mm and also equivalent to 64.8 % of the total rainfall received during this season. This is also the case for TAR B2, where the winter does not show a significant increase (1.6 %, i.e. about 11.16 mm), while a decrease of 119.5 mm (−91 %) takes place during the summer (Fig. 12). The same is observed for RCP85 and A2 scenarios; for RCP85 during the winter, rainfall decreases by about 108.7 mm (15.7 %), while it is approximately 78.8 mm (62.3 %) during the summer. The decrease is approximately 231.5 (33 %) and 113.4 mm (86 %) for the winter and summer, respectively, for A2.

The Araucanía region is only covered by two AR5 pixels, separating the region into two zones: North and South (Fig. 1). Therefore, if we were to analyze these scenarios depending on northern and southern regions, we would observe a lesser effect for RCP45 in the northern region (decreasing by about 194.4 mm, i.e. 14.8 %) than in the southern region (decreasing by about 403.1 mm per year, i.e. 24.7 %), both with respect to the corrected baseline climatology. The same is observed for RCP85, where a larger decrease in rainfall takes place along the southern pixel (−803.1 mm, approximately 49 %) than northern pixel (−92 mm, approximately 7 %). Within the TAR scenarios, the inverse patterns take place: B2 projects a decrease by about 386.35 mm per year (−29.4 %) in the North and practically no effect (even an increase of 14 mm, i.e. 0.9 %) in the southern region. For A2, the same pattern takes place, but the effect is less important: −660 mm (i.e. −50.27 %) over the northern and −592 mm (i.e. −36.21 %) over the southern pixel.

## Conclusion

Due to the expected climate change, we face the challenge of understanding what its effect will be on regional climates. IPCC projections were downscaled to higher spatial resolutions by using dynamical downscaling (Seth and Rojas 2003; Wilby et al. 2004; Conde et al. 2011). Nevertheless, these must be validated (Räisänen 2007; Refsgaard et al. 2014; Monier et al. 2014), and if necessary, be locally corrected (Bakker et al. 2014), in order to make an accurate climatic basis. Thus, from a large number of in situ time series, we first created a precipitation database to obtain a climatology. Second, we described their variability with respect to ENSO events. It was also possible to compare and correct the present historical variability produced by the model with the local reality. This correction was later applied to each of the dynamical downscaling of future projections, allowing these to properly make comparisons with the present conditions. It is important to state that the DGF PRECIS projections are the main approach currently used in Chile to define public policies under future climatic changes.

The region’s climatology shows that under Neutral (not ENSO years), an annual rainfall takes place of 1750 ± 29 mm per year with a positive gradient from the Northwest to the Southeast of about 4.8 mm km^−1^. Higher rainfall occurs during the winter, with 259 mm per month, and lower rainfall levels occur during the summer, with 53 mm per month. Months without rainfall occur with a frequency of 2.4 % and are concentrated in summer. Our climatology is consistent with older decryptions performed for this zone, such as Peña and Romero (1977) or Rouanet (1983).

Depending on the phase of the ENSO year, the amount of annual accumulated precipitation either increases (during El Niño years, by about +134 mm per year) or decreases (during La Niña years, by about −132 mm per year) in comparison to Neutral years. These effects are statistically significant (ANOVA test, 95 % of significantly level). Geographically speaking, a La Niña year is characterized by low precipitation levels in the winter in the southeast (39°S, 72°W), a region where it generally rains the most, and higher levels of precipitation in the northwestern area (37°S, 73°W). This pattern is reversed during an El Niño year.

When comparing the in situ climatology with the one from the dynamical downscaled historical run, our study shows that projections can overestimate precipitation levels for the end of the twenty-firs century (2070–2100). Thus, taking into account the rated overestimation of precipitation carried out during 1961–1991, both A2 and B2 scenarios were corrected.

After the corrections, the conservative critical scenario—in this case B2—predicts a reduction in annual precipitation of 19.19 %, equivalent to 287 ± 42 mm per year less than a current Neutral year. For the corrected A2 scenario, a decrease was predicted in annual precipitation of about −665.9 ± 27.4 (−43.8 %), less than a present-day neutral year. Seasonally, this reduction is higher during summer (86 and 91 % for A2 and B2, respectively) and is predicted to affect the whole region, except the southern region for B2. We highlight that in both scenarios, the final amount of precipitation is less than the one received on average during La Niña years from 1961 to 1991, which is a significant decrease.

The analysis of the AR5 impact confirms the DGF-PRECIS scenarios. The corrected AR5 projections show similar effects to the PRECIS with respect to in situ climatology in about −298.7 mm per year (−20.27 %) for AR5′s RCP45 and −665.9 mm per year (−43.8 %) for the RCP85. The predicted changes in precipitation will have a dramatic impact on several socioeconomic fields, especially agribusiness. For example, the combination of changes in soil–plant systems (Clark and Lynch 2009) and an increased probability of flooding (Rosenzweig et al. 2002) may cause additional crop damage. Furthermore, accelerated population growth (INE Araucanía 2015) will increase pressure on supplies for freshwater. Therefore, improved projection is crucial if the impact of climate change is to be mitigated. Correction of dynamically downscaled projections is needed, especially due to the level of bias (Baigorria et al. 2008). Few studies have been carried out in this geographical area. Performed over an area located at about the same latitude on the Pacific coast of the USA, a comparison of quality of different simulations gave good results (Doherty et al. 2003). It is noteworthy that the Hadley Center model (HadCM3) was originally selected for PRECIS downscaling because of its correct representation of South American climate variability (Seth and Rojas 2003). In addition, the new version used here for AR5 projection is the model that shows the highest correlation with Southern American rainfall (Galicia and Camilloni 2014). Finally, Hadley Center models are applied in several studies in order to model the climate impact in South America (Nóbrega et al. 2011; Cavalcanti and Shimizu 2012; Chou et al. 2014).

Lastly, since dynamically downscaled projections at a high resolution from AR5 scenarios in the region are not yet available, we do not know the impact of the dynamical physical downscaling on the amount of precipitation (as was the case with TAR DGF PRECIS). This impact could be significant since as an example, a high resolution representation of the rainfall processes in the coastal mountains and the Andes mountain range can help to get an effect on the amount of local rainfall when in situ data is unavailable locally. It is relevant to next simulate how a dynamic projection and later correction would have been. This is feasible, assuming that the dynamic downscaling performed during the TAR DGF PRECIS experience is ideally repeated with the AR5.

Thus, the TAR HadCM3 corrected scenario (original grids at 2.5) projections show 1497 mm per year (corrected baseline or in situ), 1386 mm per year (B2), and 1269 mm per year (A2). That is, a loss of 111 mm per year (7.4 %) for B2 and 228 mm per year (15.2 %) for A2. The corrected TAR DGF-PRECIS shows 1497 mm per year (corrected baseline or in situ), 1209.6 mm per year (B2), and 847.8 mm per year (A2), meaning −287 mm per year (19.19 %) for B2 and −655.9 mm per year (43.8 %) for A2. Indeed, the dynamical downscaling process doubled the amount of precipitation loss (in mm per year) for both scenarios.

Now, if we take into account the possible dynamical downscale effect for the corrected projections (using then PRECIS model) by CBQM, we would get 1127 mm per year (for RCP45) and 610 mm per year (for RCP85), a precipitation loss of −369 mm per year (24.66 %) for RCP45 and −817 mm per year (54.58 %) for RCP85. Both values are higher by 100 mm per year than the largest amount of precipitation loss obtained without dynamic downscaling, AR5, or the TAR corrected DGF-PRECIS projected scenarios (see above).

We summarize that the amount of precipitation loss over the last decades of the twenty-first century could be as high as close to 50 % of the total amount of water received, which is certainly a huge amount of water loss for regions where a large part of the activity is related to water-dependent economies (forestry, agriculture, tourism). These numbers are simply an initial guess as they should be certified by precisely effectuating a higher resolution dynamic downscaling modeling. This is a step the team is already executing, all the same, with a WRF model (Skamarock et al. 2005) instead of PRECIS. In addition, we are also comparing statistical downscaling with dynamical downscaling methodologies.

 Finally, the generated database identifies the main uncertainties and improves the current provided information for making policies and climate-change adaptation strategies. Thus, we expect that this work will be an important step to support a decision making system and design suitable countermeasures to help the Araucanía Region adapt for future climate conditions.

## Additional file

10.1186/s40064-016-3157-6 ENSO events data.

## Abbreviations

AOGCM: Atmospheric and Oceanic Global Circulation Model
AR4: Assessment Report fourth
AR5: Assessment Report fifth
CBCR: correction based on coefficient rates
CBQM: correction based on quantile mapping
DGF: Departamento de Geofísica de la Universidad de Chile. Thus DGF-PRECIS is the dynamic downscaling performed by this institution using the PRECIS model
ENSO: El Niño-Southern Oscillation
PDO: Pacific Decadal Oscillation
PRECIS: providing regional climates for impacts studies
RCP: representative concentration pathway
SST: sea surface temperature
TAR: Third Assessment Report
