# Supplementary material for: Enhanced mesoscale climate projections in TAR and AR5 IPCC scenarios: a case study in a Mediterranean climate (Araucanía Region, south central Chile)
Source: Springerplus. 2016 Sep 28;5(1):1669. doi: 10.1186/s40064-016-3157-6 (PMC5040658; doi:10.1186/s40064-016-3157-6)
Supplement: Supplementary file 1 — 10.1186/s40064-016-3157-6 ENSO events data. [file 40064_2016_3157_MOESM1_ESM.docx]

ENSO Event in base-line Period. -1 is La Niña month, 0 is Neutral Month and 1 is El Niño Month. For facility the read of the table, El Niño event are shade with a red color instead La Niña event are shade with blue color.

| **Years** | **Jan** | **Feb** | **Mar** | **Apr** | **May** | **Jun** | **Jul** | **Aug** | **Sep** | **Oct** | **Nov** | **Dec** |
| --- | --- | --- | --- | --- | --- | --- | --- | --- | --- | --- | --- | --- |
| 1961 | 0 | 0 | 0 | 0 | 0 | 0 | 0 | 0 | 0 | 0 | 0 | 0 |
| 1962 | 0 | 0 | 0 | 0 | 0 | 0 | 0 | 0 | 0 | 0 | 0 | 0 |
| 1963 | 0 | 0 | 0 | 0 | 0 | 0 | 1 | 1 | 1 | 1 | 1 | 1 |
| 1964 | 1 | 1 | 0 | 0 | -1 | -1 | -1 | -1 | -1 | -1 | -1 | -1 |
| 1965 | -1 | 0 | 0 | 0 | 0 | 1 | 1 | 1 | 1 | 1 | 1 | 1 |
| 1966 | 1 | 1 | 1 | 1 | 0 | 0 | 0 | 0 | 0 | 0 | 0 | 0 |
| 1967 | 0 | 0 | 0 | 0 | 0 | 0 | 0 | 0 | 0 | 0 | 0 | -1 |
| 1968 | -1 | -1 | -1 | -1 | 0 | 0 | 0 | 0 | 0 | 0 | 1 | 1 |
| 1969 | 1 | 1 | 1 | 1 | 1 | 1 | 1 | 1 | 1 | 1 | 1 | 1 |
| 1970 | 1 | 0 | 0 | 0 | 0 | 0 | -1 | -1 | -1 | -1 | -1 | -1 |
| 1971 | -1 | -1 | -1 | -1 | -1 | -1 | -1 | -1 | -1 | -1 | -1 | -1 |
| 1972 | -1 | 0 | 0 | 0 | 1 | 1 | 1 | 1 | 1 | 1 | 1 | 1 |
| 1973 | 1 | 1 | 1 | 0 | 0 | -1 | -1 | -1 | -1 | -1 | -1 | -1 |
| 1974 | -1 | -1 | -1 | -1 | -1 | -1 | -1 | 0 | 0 | 0 | 0 | 0 |
| 1975 | -1 | -1 | -1 | -1 | -1 | -1 | -1 | -1 | -1 | -1 | -1 | -1 |
| 1976 | -1 | -1 | -1 | 0 | 0 | 0 | 0 | 0 | 1 | 1 | 1 | 1 |
| 1977 | 1 | 1 | 0 | 0 | 0 | 0 | 0 | 0 | 1 | 1 | 1 | 1 |
| 1978 | 1 | 0 | 0 | 0 | 0 | 0 | 0 | 0 | 0 | 0 | 0 | 0 |
| 1979 | 0 | 0 | 0 | 0 | 0 | 0 | 0 | 0 | 0 | 1 | 1 | 1 |
| 1980 | 1 | 1 | 0 | 0 | 0 | 0 | 0 | 0 | 0 | 0 | 0 | 0 |
| 1981 | 0 | 0 | 0 | 0 | 0 | 0 | 0 | 0 | 0 | 0 | 0 | 0 |
| 1982 | 0 | 0 | 0 | 1 | 1 | 1 | 1 | 1 | 1 | 1 | 1 | 1 |
| 1983 | 1 | 1 | 1 | 1 | 1 | 1 | 0 | 0 | 0 | 0 | 0 | 0 |
| 1984 | 0 | 0 | 0 | 0 | 0 | 0 | 0 | 0 | 0 | -1 | -1 | -1 |
| 1985 | -1 | -1 | -1 | -1 | -1 | -1 | 0 | 0 | 0 | 0 | 0 | 0 |
| 1986 | 0 | 0 | 0 | 0 | 0 | 0 | 0 | 0 | 1 | 1 | 1 | 1 |
| 1987 | 1 | 1 | 1 | 1 | 1 | 1 | 1 | 1 | 1 | 1 | 1 | 1 |
| 1988 | 0 | 0 | 0 | 0 | -1 | -1 | -1 | -1 | -1 | -1 | -1 | -1 |
| 1989 | -1 | -1 | -1 | -1 | -1 | 0 | 0 | 0 | 0 | 0 | 0 | 0 |
| 1990 | 0 | 0 | 0 | 0 | 0 | 0 | 0 | 0 | 0 | 0 | 0 | 0 |
| 1991 | 0 | 0 | 0 | 0 | 0 | 1 | 1 | 1 | 1 | 1 | 1 | 1 |
